# Systematic Y2H Screening Reveals Extensive Effector-Complex Formation

**DOI:** 10.3389/fpls.2019.01437

**Published:** 2019-11-14

**Authors:** André Alcântara, Jason Bosch, Fahimeh Nazari, Gesa Hoffmann, Michelle Gallei, Simon Uhse, Martin A. Darino, Toluwase Olukayode, Daniel Reumann, Laura Baggaley, Armin Djamei

**Affiliations:** ^1^Gregor Mendel Institute of Molecular Plant Biology, Vienna, Austria; ^2^Iranian Research Institute of Plant Protection, Tehran, Iran; ^3^Department of Plant Biology, Swedish University of Agricultural Sciences, Uppsala, Sweden; ^4^Institute of Science and Technology Austria, Klosterneuburg, Austria; ^5^Global Institute for Food Security, University of Saskatchewan, Saskatoon, SK, Canada; ^6^Institute of Molecular Biotechnology, Vienna, Austria; ^7^Biotic Interactions and Crop Protection, Rothamsted Research, Harpenden, United Kingdom; ^8^Department of Breeding Research, Leibniz-Institut für Pflanzengenetik und Kulturpflanzenforschung (IPK), Gatersleben, Germany

**Keywords:** protein–protein interaction network, effector proteins, Ustilago maydis, plant pathogen, yeast-two-hybrid

## Abstract

During infection pathogens secrete small molecules, termed effectors, to manipulate and control the interaction with their specific hosts. Both the pathogen and the plant are under high selective pressure to rapidly adapt and co-evolve in what is usually referred to as molecular arms race. Components of the host’s immune system form a network that processes information about molecules with a foreign origin and damage-associated signals, integrating them with developmental and abiotic cues to adapt the plant’s responses. Both in the case of nucleotide-binding leucine-rich repeat receptors and leucine-rich repeat receptor kinases interaction networks have been extensively characterized. However, little is known on whether pathogenic effectors form complexes to overcome plant immunity and promote disease. *Ustilago maydis*, a biotrophic fungal pathogen that infects maize plants, produces effectors that target hubs in the immune network of the host cell. Here we assess the capability of *U. maydis* effector candidates to interact with each other, which may play a crucial role during the infection process. Using a systematic yeast-two-hybrid approach and based on a preliminary pooled screen, we selected 63 putative effectors for one-on-one matings with a library of nearly 300 effector candidates. We found that 126 of these effector candidates interacted either with themselves or other predicted effectors. Although the functional relevance of the observed interactions remains elusive, we propose that the observed abundance in complex formation between effectors adds an additional level of complexity to effector research and should be taken into consideration when studying effector evolution and function. Based on this fundamental finding, we suggest various scenarios which could evolutionarily drive the formation and stabilization of an effector interactome.

## Introduction

Molecular interactions play a central role in the disease outcome between a pathogen and their hosts. In plants, pattern recognition receptors (PRRs) on the membrane surface recognize typical damage- or pathogen-associated molecular patterns (PAMPs). Examples of well characterized PAMPs include the flg22 peptide from bacterial flagella, and the cell-wall sugar chitin from fungi and insects, which upon receptor binding lead to the activation of PAMP triggered immunity (PTI) (Jones and Dangl, 2006). To overcome PTI, pathogens secrete small molecules, termed effectors, which have evolved to suppress the host’s immune system and create a suitable environment for its development and reproductive success (Buttner, 2016; Toruño et al., 2016; Uhse and Djamei, 2018). However, some of these effectors can be recognized by nucleotide binding–leucine-rich repeat receptors (NLRs) triggering rigorous defense responses that lead to localized cell death in the infected region (Cesari, 2018). This effectively results in a molecular arms race between plants and their pathogens as they must rapidly adapt to increasingly intricate defense and infection strategies.

It is well known that proteins from both classes of the plant’s immune system—PRRs and NLRs—rely on interactions between multiple host proteins to neutralize an invading pathogen. One of the most well studied examples of interaction between PRR proteins occurs between the membrane leucine-rich repeat (LRR) receptor kinases FLS2 and BAK1 which, upon flagellin perception, heterodimerize to trigger a rapid immune response through the initiation of a phosphorylation signaling cascade (Chinchilla et al., 2007). The complexity of the interactions that occur between membrane receptors was recently addressed in a study where many of the extracellular LRR domains tested were found to be able to homo and/or heterodimerize (Smakowska-Luzan et al., 2018). In the case of NLR proteins, there have been several effector recognition mechanisms that have been found or hypothesized (Cesari, 2018). However, evidence of an NLR interaction network and its importance for immune signaling has only recently been described with “sensor” NLRs recognizing pathogenic proteins and converging to “helper” NLRs that potentiate the signaling cascade and therefore immune responses (Wu et al., 2017). For example, the recognition of AvrAC from *Xanthomonas campestris* pv. *campestris* causes the uridylation of PLB2 which in turn binds to an NLR from *Arabidopsis thaliana*, ZAR1. This binding results in the pentamerization of PLB2 that ultimately leads to pathogen resistance (Wang et al., 2019). Altogether, the complexity of these protein receptor interaction networks resulted as a direct consequence of the diverse signals that plants integrate and coordinate to adequately respond to the challenges imposed by their environment.

*Ustilago maydis* is a biotrophic fungal pathogen able to infect all aerial parts of maize plants. Its lifestyle is supported by absorbing nutrients from sink tissues, where it induces the formation of galls and develops spores. Like other pathogenic organisms, *U. maydis* relies on effectors to perform a wide range of tasks, from host defense suppression to manipulation of plant metabolism and development to favor the pathogen’s own growth and proliferation. Although hundreds of putative effector proteins are encoded in the *U. maydis* genome, only a few of these have been functionally characterized. Examples include Pep1, which reduces the accumulation of H_2_O_2_ in the apoplastic space (Doehlemann et al., 2009), Pit2, which inhibits apoplastic cysteine proteases (Mueller et al., 2013), Rsp3, which coats the fungal hyphae preventing the activity of antifungal proteins (AFP) 1 and 2 (Ma et al., 2018), and Cmu1 and Tin2, which were proven to interfere with the production of salicylic acid and lignin, respectively (Djamei et al., 2011; Tanaka et al., 2014). Other virulence factors, such as Stp1, ApB73, and Cce1 were shown to play a role during infection, yet their functions remain elusive (Schipper, 2009; Stirnberg and Djamei, 2016; Seitner et al., 2018). While these studies expanded our knowledge of the mechanisms of biotrophic pathogenesis in plants, considering that the *U. maydis* genome encodes for many putative effector proteins it is clear that the complexity of the host–pathogen interaction is still poorly understood.

The most recent analysis of the *U. maydis* genome identified 467 proteins that are predicted to be secreted, representing almost 7% of its total proteome. Of these, 203 (43%) lack predicted domains which could indicate their function (Schuster et al., 2018). A recent comprehensive transcriptome analysis of *U. maydis* throughout its biotrophic development showed three discrete, tightly regulated expression patterns of these secreted proteins (Lanver et al., 2018). Additionally, there are effectors that are known to have tissue-specific functions. For instance, See1 was linked to DNA synthesis reactivation in the host and is essential for gall formation in leaves but not in floral tissues, where cell division occurs regardless of the infection process (Redkar et al., 2015; Matei et al., 2018). Thus, the localized and temporal regulation of effector protein expression throughout the infection process is crucial for the fungal pathogen to successfully complete its lifecycle.

Considering their relatively limited number of effectors, it is astonishing that biotrophs can overcome the highly complex host immune system and regulate biotrophic virulence in a multicellular host. An interaction network between effectors could provide an additional level of complexity to create a versatile and robust effectome. In fact, few cases of functional characterization of effector homo- and heterodimers from bacteria, oomycetes, and fungi have been reported (Gürlebeck et al., 2005; Djamei et al., 2011; van Damme et al., 2012; Flayhan et al., 2015; Ma et al., 2015; Li et al., 2016). Some of these dimers have even been linked with pathogenicity. For example, the effector PsCRN63 from *Phytophthora sojae* is only able to suppress cell death associated with PTI upon dimerization (Li et al., 2016). Other cases of bacterial effector interplay in the context of function redundancy, antagonistic effects, and in host regulation have been reported and reviewed in Shames and Finlay (2012). Despite this, there have only been a few attempts to systematically characterize interactions within a pathogen’s effector repertoire. A screen for metaeffectors (*i.e.* “effectors of effectors”) in *Legionella pneumophila* revealed 23 effector pairs with antagonistic effects in yeast cell growth, 10 of which showed direct effector–effector interaction in a yeast-two-hybrid (Y2H) setting (Kubori et al., 2010; Urbanus et al., 2016).

The relevance of protein interactions in plant immunity, the rapid co-evolutionary arms race in plant–pathogen interactions, and the increased versatility that emerges from intermolecular networks suggest that effector dimerization and complex formation could have evolved to improve fitness in *U. maydis*. Using a systematic Y2H approach, we show that homo- and heterodimerization of putative effectors is not only possible but occurs abundantly in the *U. maydis* effectome. These interactions were found between more than a third of all effector candidates tested and analyzed in context of other publicly available data to speculate on how they can affect the functionality of an effectome. Our data shed new light on how fungal effectors can act *in planta* and future functional analyses will need to take into account inter- and intraspecies protein–protein interactions, to advance our understanding of how effectors shape the infection process.

## Materials and Methods

### Strains, Plasmids, and Culture Conditions

DNA manipulation and plasmid generation were performed according to standard molecular cloning procedures (Ausubel et al., 1987; Sambrook and Russell, 2006). All DNA manipulations were performed using the *Escherichia coli* MACH1 strain (Thermo Fisher Scientific, Waltham, MA, USA). Plasmids and primers are compiled in **Tables S1**– **S3**. Some plasmids were generated using the GreenGate system (Lampropoulos et al., 2013). All vector maps containing detailed sequence information are available upon request.

The library of putative effectors was cloned based on the effector prediction analysis described in Mueller et al. (2008). The full list of genes used in this study is compiled in **Table S1**, which includes the gene specific primer sequences used to isolate the genes and the updated signal peptide prediction scores by SignalP v5.0 (Armenteros et al., 2019). All putative effectors were cloned without the predicted signal peptide encoding region into a modified pEntry4b vector either by BspHI–NotI or by NcoI–NotI restriction sites. Prior to pEntry4b cloning, native BspHI, NcoI, and NotI sites in putative effector coding sequences were mutated without affecting the encoded amino acid. The effector candidate-containing pEntry vectors were used to subclone by LR-reaction all putative effectors into the respective modified pGBKT7 and pGADT7 gateway destination vectors (Thermo Fisher Scientific, Waltham, MA, USA).

### Yeast Work

*Saccharomyces cerevisiae* strain AH109 was transformed with pGBKT7 derivatives as previously described (Rabe et al., 2016), using standard protocols (Clontech/Takara Bio, Saint-Germain-en-Laye, France). Strains carrying N-terminal Gal4 DNA binding domain (BD) fusions with putative effectors were tested for autoactivity by growth in minimal synthetic defined (SD) dropout medium and spotted on SD plates depleted of tryptophan, adenine, and histidine (SD-Trp/Ade/His). SD-Trp plates were used as a control for strain viability. pGADT7 derivates with N-terminal Gal4 activation domain (AD) fused to putative effectors from *U. maydis* were transformed into the yeast strain Y187 from the Matchmaker™ GAL4 Two-Hybrid System (Clontech/Takara Bio, Saint-Germain-en-Laye, France).

Yeast strains AH109 containing one of the 274 non-autoactive pGBKT7-effectors were mated with a library of the Y187 yeast strains containing 297 AD-effector candidate fusions. Mating was performed according to the manufacturer’s protocol. Diploids carrying both plasmids were selected on SD plates depleted of tryptophan and leucine (SD-Trp/Leu), and dimerization was tested by growth on intermediate (SD-Trp/Leu/His) and high (SD-Trp/Leu/Ade/His) stringency media. 710 colonies from SD-Trp/Leu/Ade/His plates were picked for prey identification and bait confirmation by Sanger sequencing, after colony PCR using standard protocols (Clontech/Takara Bio, Saint-Germain-en-Laye, France).

One-on-one screening was performed in liquid cultures using a Bravo Liquid Handling Platform (Agilent, Santa Clara, California, USA). *S. cerevisiae* strains carrying the 63 non-autoactive pGBKT7 strains that showed interactions in the first screen and all the pGADT7 constructs were grown in liquid SD-Trp or SD-Leu, respectively, before being co-inoculated in PD medium and left overnight to mate. This and all subsequent steps were performed in 96-well tissue culture plates (VWR, Radnor, Pennsylvania, USA). The cultures were moved to SD-Trp/Leu for 1 day to select for successful mating after which the cultures were inoculated in SD-Trp/Leu, SD-Trp/Leu/His, and SD-Trp/Leu/His/Ade and grown for 3–4 days. To determine culture growth, OD_600nm_ was measured with a Synergy 2 automated plate reader (BioTek, Winooski, VT, USA). Mating success was measured by growth on SD-Trp/Leu and successful interactions were defined as growth on all three auxotrophic media above a specified OD_600nm_ threshold: 0.1 for SD-Trp/Leu and 0.25 for the other media.

The workflow and the list of tested putative effectors for the Y2H work can be found in **Figure S1**, and **Tables S1** and **S2**, respectively.

### Transient Expression in *Nicotiana benthamiana* and Co-Immunoprecipitation

Effector candidates from a small interaction subnetwork (in focus in **Figures 2** and **S2**) were cloned into plant expression vectors by golden gate cloning using the vectors and the methods described in Lampropoulos et al. (2013). Expression of the putative effectors was controlled by the strong 35S promoter and N-terminally tagged with either HA or C-myc triplicated sequences. All vector maps containing detailed sequence information are available upon request. The vectors were then electroporated into the *Agrobacterium tumefaciens* strain GV3101 and infiltrated into leaves of 4 to 5 week-old tobacco plants. Three days post-infiltration, the plant material was harvested, snap frozen in liquid nitrogen, milled using a Retch Mixer Mill MM 400 (Retsch GmbH, Germany) at 30 Hz for 1 min 30 s, and kept at −80°C until further processing.

The plant powder was resuspended in IP buffer (50 mM HEPES pH 7.5, 100 mM NaCl, 10% glycerol, 1 mM EDTA, 0.1% Triton X-100, 1 mM PMSF, 2% PVPP, and protease inhibitor cocktail) and centrifuged three times at 20,000×*g* to remove solid debris. C-myc tagged proteins and their interacting proteins were isolated using the µMACS c-myc Isolation Kit (Miltenyi Biotec, Germany) following the manufacturer’s manual, using the above described buffer without PVPP for the washes. For Western Blot protein detection, samples were resolved by SDS-polyacrylamide 4–20% gradient gel electrophoresis and transferred to a nitrocellulose membrane using the Trans-Blot Turbo Transfer System (Bio-Rad, CA, USA). C-myc tagged proteins were probed with a mAB α-Myc-tag; clone 9E10 (produced by the Molecular Biology services from the GMI/IMBA/IMP service facilities) and detected by hybridizing with a sheep raised anti-mouse secondary antibody coupled to horseradish peroxidase (HRP; GE Healthcare, IL, USA). HA tagged proteins were detected using the HRP coupled anti-HA antibody raised in mouse (Miltenyi Biotec, Germany). HRP activity was visualized by using the SuperSignal^™^ West Pico PLUS Chemiluminescent Substrate (Thermo Fisher Scientific, MA, USA) and imaged in either a ChemiDoc imaging system (Bio-Rad, CA, USA) or on Amersham Hyperfilms ECL (GE Healthcare, IL, USA), depending on protein amounts.

### Data Handling and Network Analysis

Data from the various experiments were combined and analyzed using R scripts (R Core Team, 2014). The network analysis and visualization were done using the open-source, JavaScript-based graph library Cytoscape (Franz et al., 2016).

To check that the interactions were not occurring randomly, we calculated the number of interactions which would have been observed in triplicate given the number of interactions detected in each replicate of the screen if the growth were random. We then used the Fisher’s exact test to compare the expected and observed number of interactions to see whether our results differed from random growth.

## Results and Discussion

### Many Putative Effectors From *U. maydis* Are Highly Interconnected

To identify effectors able to homo- or heterodimerize and to estimate their abundance, we cloned 310 putative effectors of *U. maydis* without their predicted signal peptides into Y2H vectors encoding either an N-terminal Gal4 DNA binding domain (BD; pGBKT7 derivate) or an N-terminal Gal4 activation domain (AD; pGADT7 derivate). After excluding autoactive strains, each of the remaining 274 bait strains were mated against a pool of 297 AD-effector candidate prey strains and incubated on high selection plates. Colony growth was observed on 86 plates. Thus, at least 30% of the putative effectors tested were able to interact with each other in the Y2H. Despite sequencing the pGADT7 inserts from 710 yeast colonies we did not reach screen saturation, revealing that the effector candidate interaction network is more complex than previously thought.

**Figure 1:**
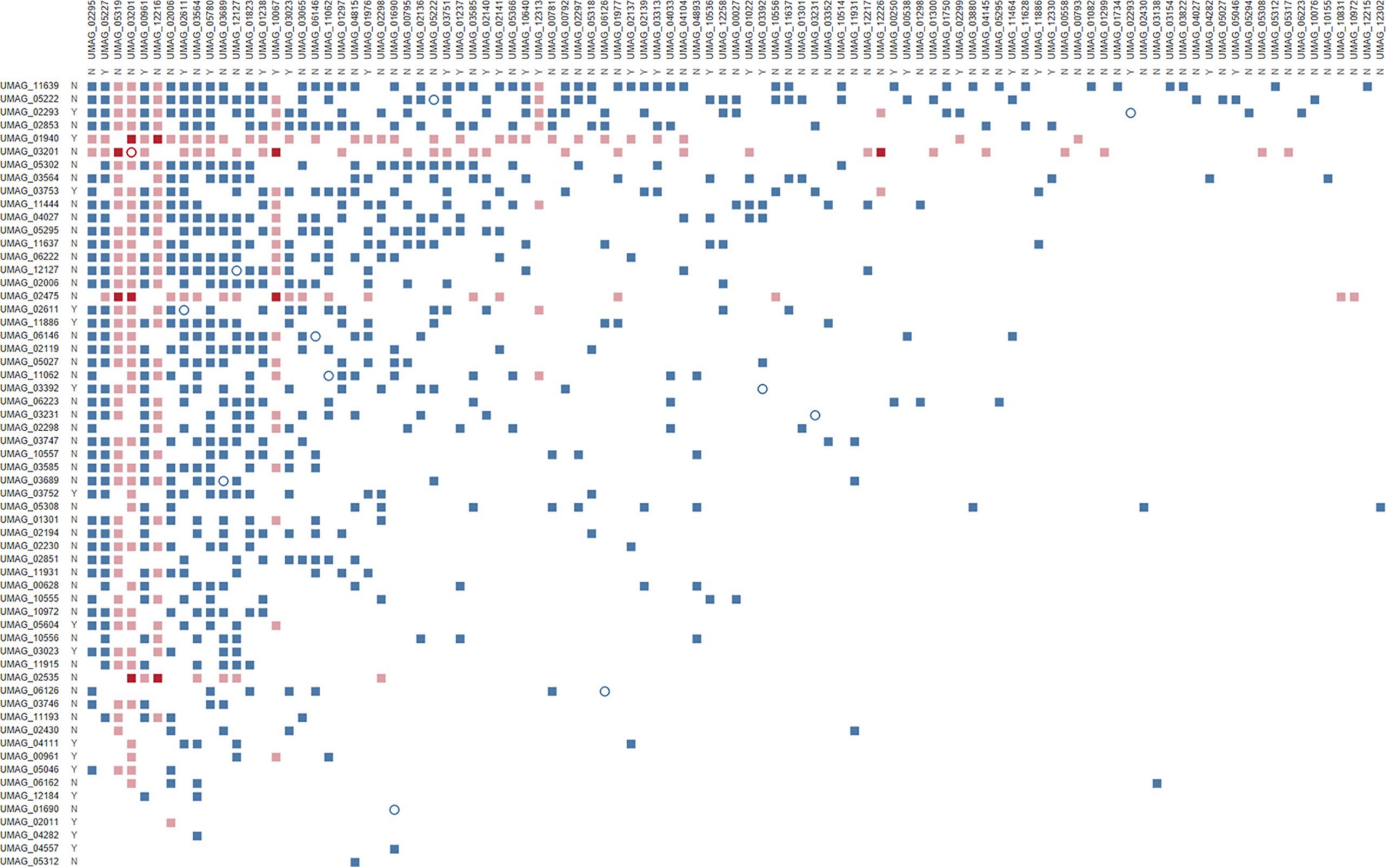
Effector–effector interactome of *U. maydis* confirmed by three independent Y2H replicates. Bait N-terminal Gal4 DNA binding domain (BD) fusions with putative effectors are listed on the left, while prey N-terminal Gal4 activation domain–effector fusions are on the top. Both axes are ordered by decreasing number of interactions. Letters indicate whether proteins are core (Y) or non-core (N) effectors (Schuster et al., 2018); full squares represent heterodimers, while empty circles show homodimers; colors represent effectors that show virulence defects upon deletion in either both (red), one (pink), or none (blue) of the effector pairs (Uhse et al., 2018).

To overcome this issue, we used a robotic system to perform individual matings in liquid medium between the 86 bait putative effectors that showed interactions on plate with each of the 297 AD-effector candidate fusions. This change in methodology led to a reduction in screened bait proteins to 63, either due to technical issues or because the strains exhibited some growth in high-selection liquid medium before mating. This change in autoactivation was most likely caused by the known difference of growth rates in liquid *vs* solid media (Herricks et al., 2017), and by having an OD_600nm_-based threshold for the liquid cultures rather than a subjective visual inspection of growth on plates. With this final protein set, bait putative effectors were transformed and mated independently three times. Interactions with prey strains were only considered valid if they were reproduced in all three matings. This resulted in an interaction matrix of 126 putative effector proteins producing 867 unique potential interactions (**Figure 1**), representing a highly connected network of protein interactions between almost 40% of the tested proteins. The number of interactions per putative effector varies between 1 (for 26 putative effectors) and 63 (in the case of UMAG_03201), and 12 putative effectors showed the ability to form homodimers. In the few instances where interactions were tested reciprocally, we observed 26 interactions where the swap of bait and prey domains did not influence the observed interaction. Altogether, this highly connected effectome can lead to increased versatility and robustness in the context of the molecular arms race between plants and their pathogens.

By integrating publicly available datasets with the interaction matrix, we identified other emerging features of this interactome regarding genome clustering, relevance in infection, and conservation in closely related species. More than 18% of the putative effector encoding genes from *U. maydis* are clustered in the genome (Kämper et al., 2006) and it has been observed that co-localization in these genomic islands allows for transcriptional co-regulation and might implicate involvement in similar biological processes. However, our network has only 61 interactions between putative effectors encoded in the same chromosome that are on average approximately 350 kbp apart, therefore not showing a bias for effector candidates to interact with others within the same cluster. Uhse et al. (2018) established a next-generation sequencing-based screening method that enabled the identification of new virulence factors of *U. maydis*. Ten of the 28 virulence factors identified in that screen showed potential interactions with multiple other putative effectors (**Figure 1**). This could explain why these proteins are important for virulence as multiple other effectors might be in a complex with them to fulfil their roles during infection. Finally, when looking at conserved effectors between closely related pathogens (*i.e.* core effectors; Schuster et al., 2018) there was no overrepresentation of interactions between only core or non-core effectors (**Figure 1**). This means that effector–effector interactions are not abundantly conserved among smut fungi and, therefore, result from adaptation to specific host–pathogen interactions. Nevertheless, it would be interesting to address the relevance of the interactions between any given core effector pair by testing whether interactions are also formed between the orthologs and are therefore a conserved feature. This could help focus functional studies of effector–effector interactions on those with higher likelihood of biological significance.

Although Y2H assays have been widely used to identify host protein-effector interactions and enabled significant advances in the field of effector biology (Mukhtar et al., 2011; Weßling et al., 2014), the methodology has its limitations as it forces co-expression and co-localization of the two proteins tested for interaction. This limitation may result in some false positives among the interactions found. However, it is important to note that based on the number of interactions detected in each of the three screens, if the interactions were to occur purely by chance we would expect only 48 interactions to be confirmed across the three replicates. Instead, we found 867 interactions, which is significantly more than if the observed interactions were to occur randomly (p = 1.16 × 10^−200^). This increases the confidence in these results and indicates that interactions between proteins from the *U. maydis* putative effectome are seemingly highly complex (**Figure 1**). On the other hand, considering the exclusion of putative effectors with autoactivity from our screen and that the Gal4 activation and DNA-binding domains may interfere with the ability of some putative effectors to interact, it is likely that some meaningful interactions were not detected and the effector candidate interactome presented here might still be underestimated.

Attempts to confirm some of these interactions during infection proved to be extremely difficult due to the low concentration of specific putative effectors as a result of the relatively insignificant fungal biomass in comparison to the maize tissue. In fact, RNA-seq data from maize infected tissue showed that fungal RNA represents less than 5% of total transcript abundance at 8 days post infection (dpi; Lanver et al., 2018). In order to not disturb the fine balance of protein expression and therefore their interactions, our efforts focused on using knockout strains of specific putative effectors complemented by in-locus recombination of tagged versions under control of the endogenous promoters. Western blots from co-immunoprecipitation samples of infected tissues proved to be below the detection threshold for the specific interactions tested (data not shown). Therefore, an improved method of protein detection from infected tissue will be needed to independently validate the interactions in maize.

**Figure 2:**
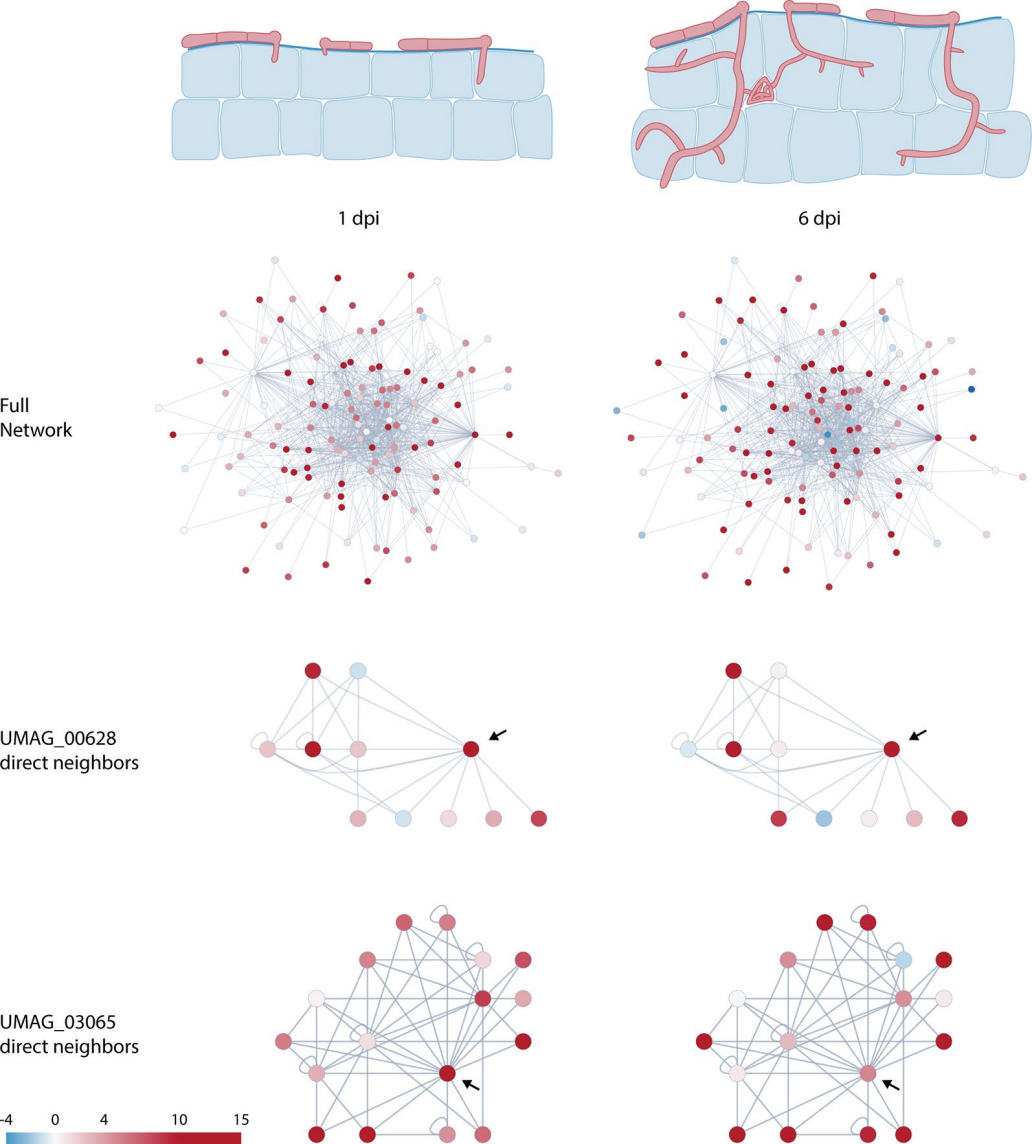
Schematic representation of *U. maydis* infection at 1 and 6 days post infection (dpi) and changes in predicted protein–protein interaction network dynamics of putative effectors. Nodes are colored according to Log_2_ fold change relative to axenic culture (Lanver et al., 2018). Arrows in the subnetworks indicate network centers.

Regardless of the mentioned limitations, we took a subset of one of the subnetworks shown in **Figures 2** and **S2**, and tried to confirm 12 of its interactions using an alternative method. Protein pairs with either an HA or c-myc triple tag were transiently co-expressed in *N. benthamiana*, followed by co-immunoprecipitation (co-IP, **Figure S3**). We used the c-myc tagged proteins as bait and a c-myc tagged mCherry construct as a negative control to exclude the possibility of false positive interactions from technical constraints (samples 1, 2, and 3). Within the subnetwork, several interactions found previously by Y2H could be confirmed (namely for the interactions between UMAG_03201 and UMAG_03689, UMAG_05227 and UMAG_03564, UMAG_05780 and UMAG_03689, UMAG_05780 and UMAG_03201, and UMAG_03689 and UMAG_03564). The results for the remaining tested interaction pairs did not overlap with the Y2H data which could be due to inherent limitations in both techniques. For instance, in both cases, the proteins are not secreted as they would be in the native system and are expressed with tags that can differently interfere with their stability, solubility and function. While both methods rely on the heterologous expression of proteins, the yeast expression system is phylogenetically closer to *U. maydis*, which potentially influences expression of some proteins and could be one reason for the observed discrepancies between the results. The major conclusion from integrating the information obtained from both, the Y2H and the co-immunoprecipitation approach, is that a great number of effectors should not be characterized in isolation but instead in context with their respective potential interacting co-effectors. The characterization of all interactions would be well beyond the scope of this study, but the integration of the network we identified opens the possibility to further test single interactions and represents a valuable resource for future effector research.

### Change in Expression Profiles Throughout Infection Reveals Network Dynamics

Effector expression is tightly regulated and commonly occurs in waves during the course of infection (Toruño et al., 2016). These expression profiles enable the pathogen to finetune the plant’s defense and metabolism along its lifecycle to create a favorable environment for its development. By combining our data with the recently published RNA-seq data of infected maize leaves at different timepoints (Lanver et al., 2018) we were able to see how interactions might change during the infection process. As effectors are expressed at different levels during the infection process, they may form different dimers or even complexes with different functions throughout the biotrophic stages of the pathogen. **Figure 2** shows how expression levels change within the global network at two different infection stages: 1 and 6 dpi.

By focusing on specific proteins, this network plasticity becomes more obvious. For instance, UMAG_03065 interacts directly with 10 other putative effectors, with only two of them not being expressed in the earlier infection stage (**Figures 2** and **S2**). However, at the later timepoint, more protein coding sequences within this network seem to be downregulated, resulting in fewer possible interactions. In the case of UMAG_00628, the central protein in this network has lower expression at 6 dpi, opening the possibility for the peripheral proteins in this subnetwork to interact with each other (**Figures 2** and **S2**). Both examples highlight the changing interaction network and suggest an additional level of plasticity in the *U. maydis* effectome from the interplay between gene expression patterns and protein–protein interactions. In addition to gene expression, this plasticity can be further increased by the affinity of the interaction. Given different interaction partners, more dimers will be formed between the proteins that have a higher binding affinity. Finally, effectors that are translocated through different compartments are subjected to different conditions (*e.g.* pH) that can change the affinity of two proteins to bind to each other. Thus, depending on the subcellular localization of the proteins in the network their affinity, and therefore their function, can be affected.

It is important to note that this overlay of interaction with expression data can reveal observed interactions that might not relate to a biological function, in cases where a protein pair does not show co-regulation during the life cycle of *U. maydis*. However, it is equally relevant to mention that other factors influence effector gene expression, such as host tissue specificity. Using microarrays, it was found that at 3 dpi only 21% of upregulated *U. maydis* effector genes were expressed in three different maize tissues while 45% were expressed in only one type of tissue (Skibbe et al., 2010). Therefore, some of the interactions found here are probably relevant in a tissue specific context, rather than the infection stage.

### Functional Models of Effector Interactions

**Figure 3:**
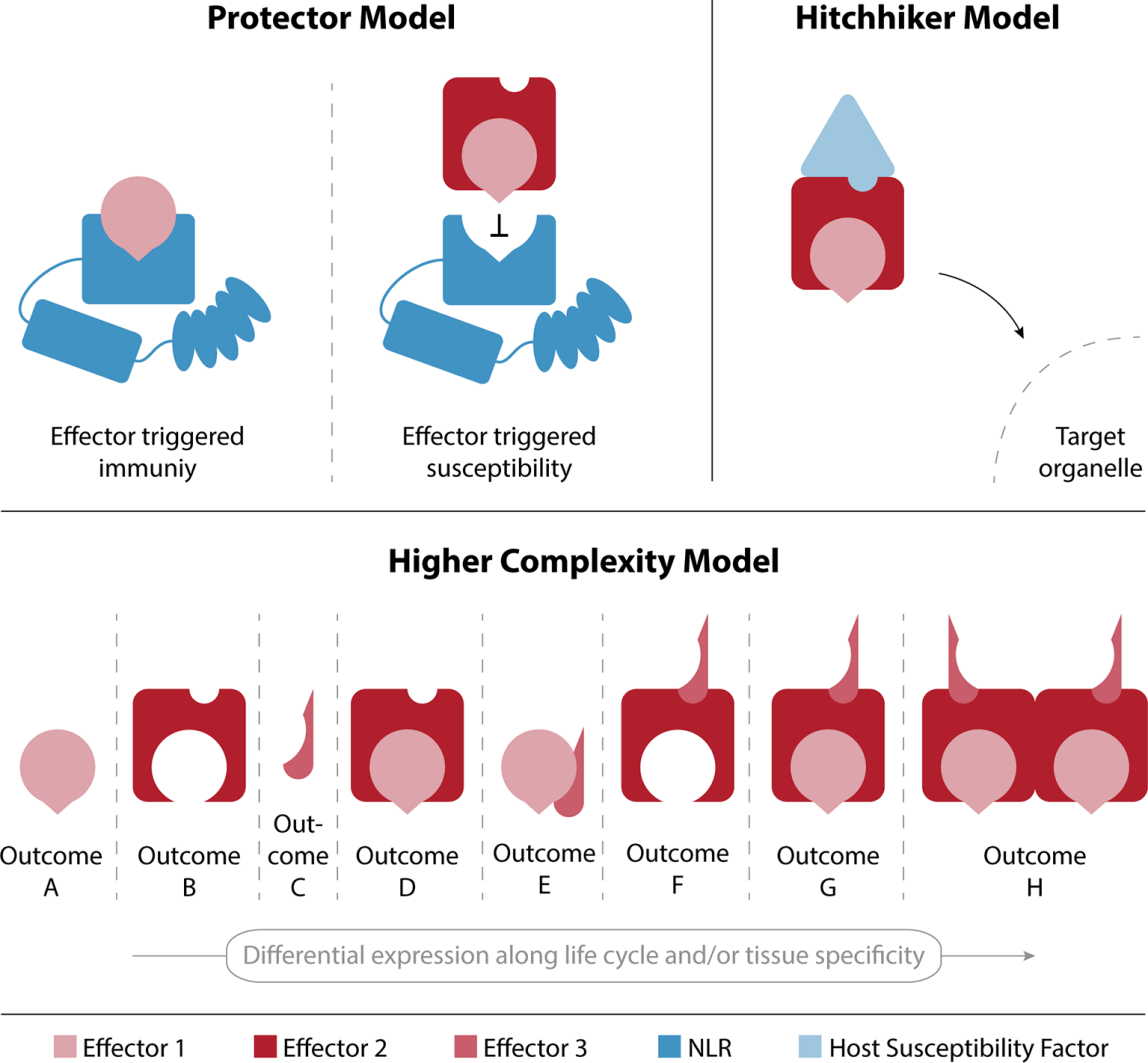
Models for effector-effector interaction outcomes. The protector model describes an interaction between effectors 1 and 2, which results in the avoidance of recognition of effector 1 by the plant’s immune system and therefore leads to a successful infection. In the hitchhiker model, effector 1 is able to shuttle to its target organelle by interacting with effector 2, which in turn interacts with a plant susceptibility factor that mediates the shuttling upon effector binding. The higher complexity model highlights the plasticity that can emerge by differential effector expression along the pathogen’s lifecycle and/or in specific tissues.

There are many evolutionary driving forces that can lead to the stabilization of effector–effector interactions. In **Figure 3** we speculate on a few possible outcomes from interactions between effectors and propose three possible models. Plants evolved NLRs for direct or indirect effector recognition leading to effector-triggered immunity (ETI; Jones and Dangl, 2006; Macho and Zipfel, 2014; Wu et al., 2017). It is therefore feasible that effectors have evolved to interact and compete for receptor recognition sites that would activate ETI, in what is referred to as the “protector model” in **Figure 3**. In this example, effector 1 is recognized by a plant NLR and will trigger ETI. However, upon interaction with effector 2, the site that the plant NLR recognizes is blocked and the pathogen can continue the infection process. This mechanism could also lead to the protection of effectors from plant proteases or other possible protein modifications that would impede their function or target them for degradation.

Another possibility would be that an effector interacts with the host cellular machinery to shuttle with it to a specific subcellular compartment and fulfil its biological role. The “hitchhiker model” represents this effector as an interaction hub with other effectors that can shuttle with it to the same subcellular destination. In the representation given in **Figure 3**, the localization of effector 2 is determined by its interaction with a plant susceptibility factor, and its ability to bind to other effectors—such as effector 1—results in the shuttling of multiple effectors to a target organelle in host cells. The relevance of interaction-dependent protein localization to subcellular compartments is well known and has even been shown to play an important role in a similar interactome dataset of endosomal sorting complexes, required for transport (ESCRT) proteins in *A. thaliana* (Richardson et al., 2011). This mechanism would allow for the evolution of a very efficient transporter that acts as a hub for effector shuttling, rather than having localization signals in all effector proteins.

Finally, the presence of different effectors can lead to different outcomes depending on their spatial and temporal distribution. **Figure 2** shows the plasticity of interaction networks that can be created along the infection process as a direct consequence of effector expression patterns. It is reasonable to assume that the interactions between effectors described here, lead to an increased phenotypical complexity shown in the combinatory model of **Figure 3**. The illustration of the “higher complexity model” shows how outcomes can vary by changing the expression of three proteins either in different tissues or along the pathogen’s life cycle. This model has recently been suggested by Thordal-Christensen et al. (2018), and the overlay of our data with the publicly available expression profile supports such a scenario. The exact nature of those outcomes remains to be determined and further research is needed to shed new light into the full extent of the plasticity that interactions between effector proteins can confer.

The network presented here is neither complete nor will all Y2H verified interactions play a biological role. Nevertheless, it provides a valuable framework for future *U. maydis* effector studies and widens our view on the consequences of the co-evolution between the host immune system and the effectome of the pathogen. The extent of the interactome shows that effector biology is more complex and intricate than previously thought and the possibility of effector–effector interactions should not be neglected when studying plant–pathogen interactions.

## Conclusion

Protein–protein interactions are crucial for diverse biological functions across all lifeforms. While increasing evidence suggests extended protein interaction networks among plant immune receptors, little focus has been put on protein interactions between virulence factors that have co-evolved with it. Here we show evidence of complex effector–effector interactions in *U. maydis* that seem to mirror the intricate networks found in plant immune systems. Despite the limitations of the Y2H methodology, the *U. maydis* effectome shows a surprisingly high number of interactions between secreted proteins. In combination with temporal and spatial regulation, future functional characterization of effectors will need to take into consideration the possibility of effector–effector interactions and their role in the infection process.

## Data Availability Statement

All datasets for this study are included in the article/**Supplementary Material**.

## Author Contributions

AD conceived the original research plan and designed the experiment. AA, JB, FN, GH, MG, SU, MD, TO, DR, and LB contributed to the experimental work. JB coordinated the experimental work and tested the interactions. JB and AA did the data analysis. AA, AD, and JB wrote the manuscript.

## Funding

This work was supported by the European Research Council under the European Union’s Seventh Framework Programme (FP7/2007-2013)/ERC grant agreement n8 (GA335691 “Effectomics”), the Austrian Science Fund (FWF) (I 3033-B22, P27818-B22), and the Austrian Academy of Science (OEAW).

## Conflict of Interest

The authors declare that the research was conducted in the absence of any commercial or financial relationships that could be construed as a potential conflict of interest.

## Supplementary Material

Table S1 List of putative effectors used as either bait and/or prey proteins. Gene specific primer sequences used to clone the coding sequences, signal peptide prediction scores, and peptide length are also listed.

Table S2 Identity of bait proteins used in the liquid media screen. Baits were tested for autoactivity by growth in liquid culture and on plates. In the mating test (SD-Leu/-Trp/-His/-Ade) the bait strain was mated with yeast containing an empty pGADT7 plasmid. In liquid tests, strains were considered autoactive when OD600 was higher than 0.25 after 3 days of incubation at 28oC.

Table S3 Primers used for sequencing of inserts (effectors) after gateway reaction.

Figure S1 Workflow of Y2H work. Numbers represent coding sequences of putative effector proteins tested. See **Supplementary Tables S1**, **S2**, and **S3** for further details.

Figure S2 Detailed description of the subnetworks represented in **Figure 2**. Core effectors were identified in Schuster et al. (2018), clusters were described in Kämper et al. (2006), iPool-Seq data was obtained from Uhse et al. (2018), and sequencing data was taken from Lanver et al. (2018). The centers of the networks are highlighted in bold; circles represent homodimers and squares represent heterodimers.

Figure S3 Co-immunoprecipitation of 12 proteins from the UMAG_00628 subnetwork. Proteins were tagged with either 3x myc or 3x HA N-terminal tags, which was the same side of the activation and binding domains in the Y2H screen. Nicotiana benthamiana plants were transiently transformed and expressed the fusion proteins for 3 days before harvest. On the left, the interactions found by Y2H in the subnetwork subset are illustrated. Full blue boxes with white numbers represent expected interactions, empty boxes with black numbers represent protein pairs that are not expected to interact, and circles represent homodimers. On the right are the same interactions tested by Co-immunoprecipitation. The sample numbers from the Y2H matrix for each interaction pair are represented on top of the western blots.
