# Supplementary material for: Systematic Y2H Screening Reveals Extensive Effector-Complex Formation
Source: Front Plant Sci. 2019 Nov 14;10:1437. doi: 10.3389/fpls.2019.01437 (PMC6872519; doi:10.3389/fpls.2019.01437)
Supplement: Figure S1 — Workflow of Y2H work. Numbers represent coding sequences of putative effector proteins tested. See Supplementary Tables S1, S2, and S3 for further details. [file Presentation_1.pptx]

## Slide 1
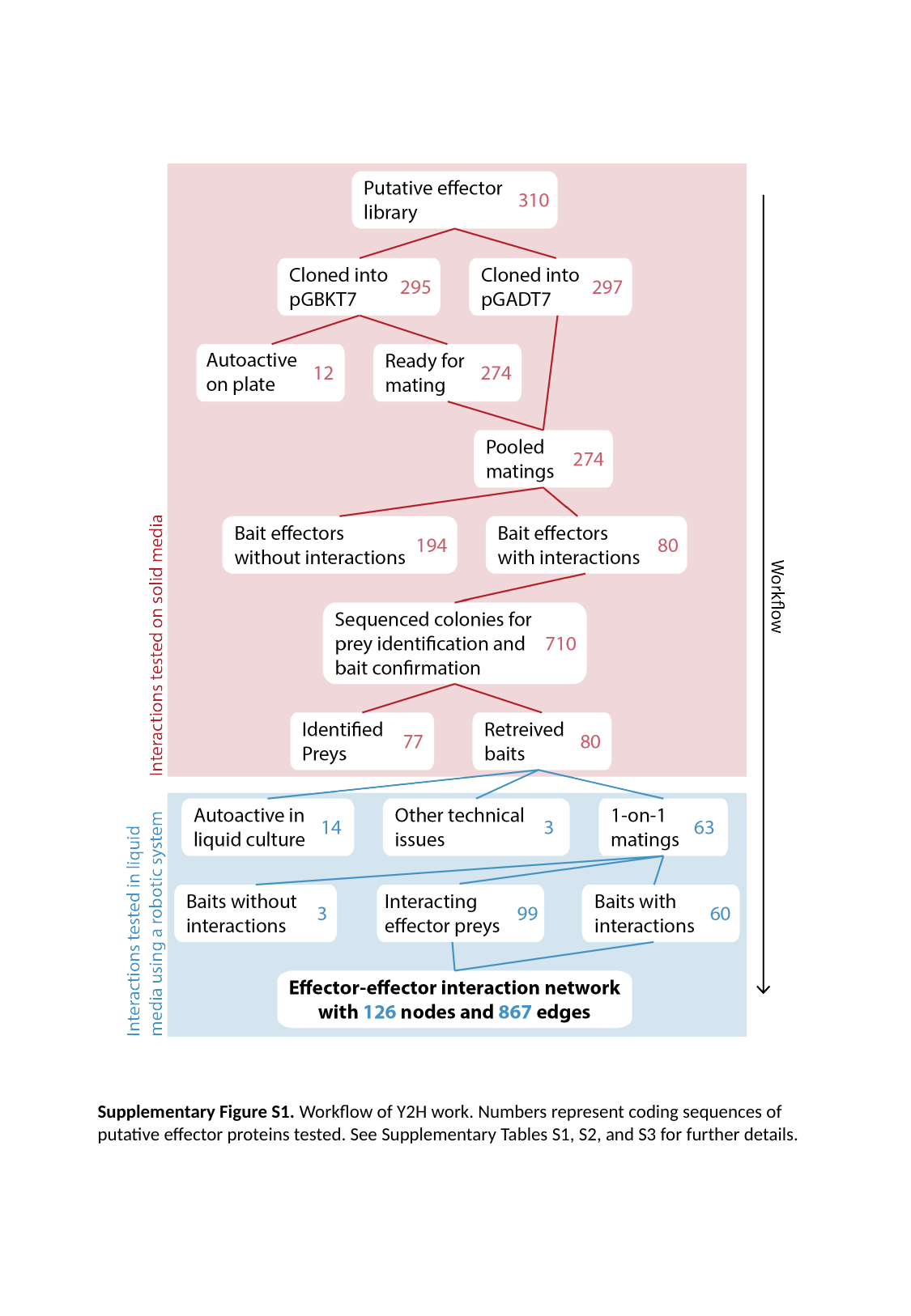

Supplementary Figure S1. Workflow of Y2H work. Numbers represent coding sequences of putative effector proteins tested. See Supplementary Tables S1, S2, and S3 for further details.

## Slide 2
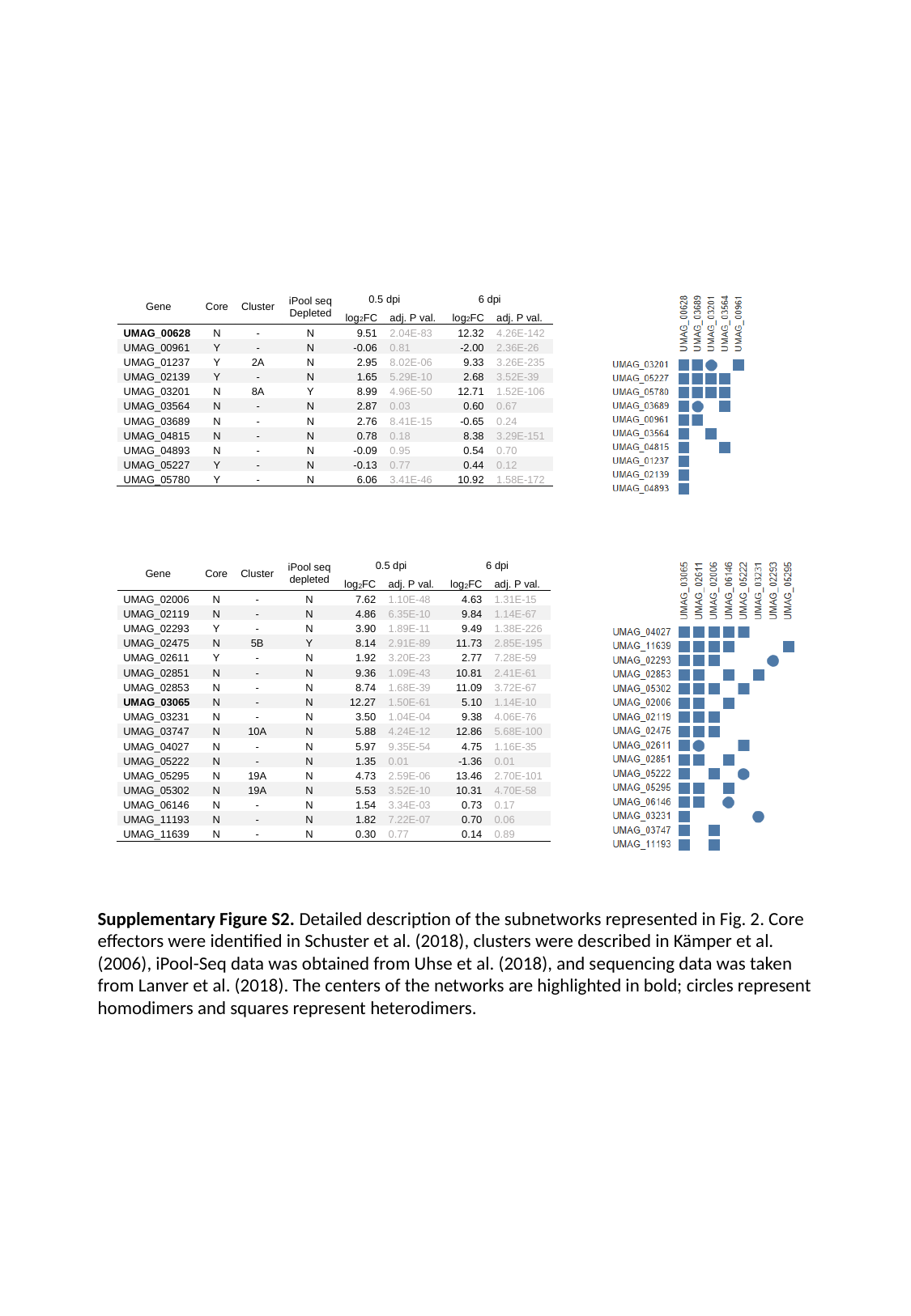

Supplementary Figure S2. Detailed description of the subnetworks represented in Fig. 2. Core effectors were identified in Schuster et al. (2018), clusters were described in Kämper et al. (2006), iPool-Seq data was obtained from Uhse et al. (2018), and sequencing data was taken from Lanver et al. (2018). The centers of the networks are highlighted in bold; circles represent homodimers and squares represent heterodimers.

## Slide 3
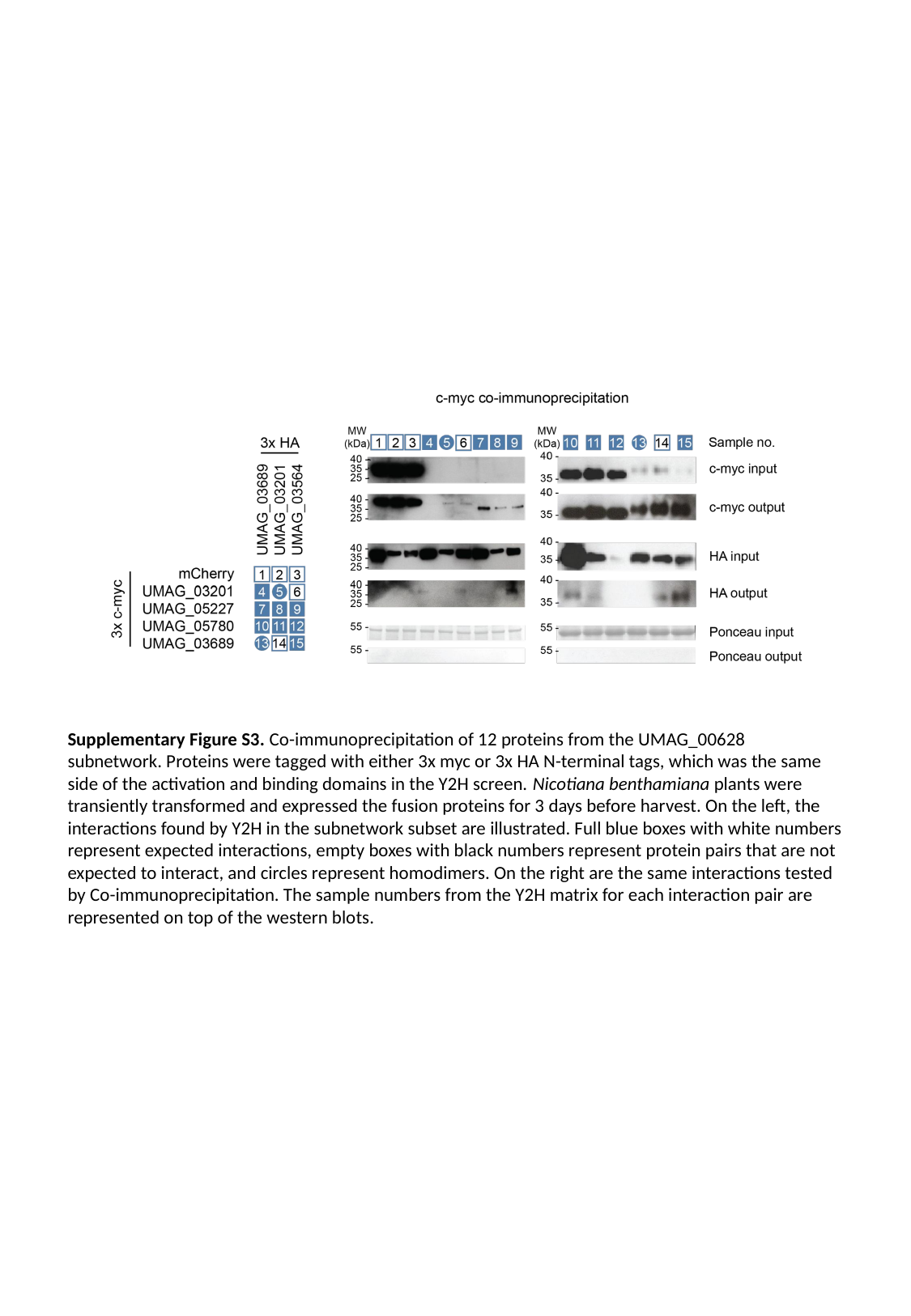

Supplementary Figure S3. Co-immunoprecipitation of 12 proteins from the UMAG_00628 subnetwork. Proteins were tagged with either 3x myc or 3x HA N-terminal tags, which was the same side of the activation and binding domains in the Y2H screen. Nicotiana benthamiana plants were transiently transformed and expressed the fusion proteins for 3 days before harvest. On the left, the interactions found by Y2H in the subnetwork subset are illustrated. Full blue boxes with white numbers represent expected interactions, empty boxes with black numbers represent protein pairs that are not expected to interact, and circles represent homodimers. On the right are the same interactions tested by Co-immunoprecipitation. The sample numbers from the Y2H matrix for each interaction pair are represented on top of the western blots.
